# Voices From the Outbreak: Exploring the Role of Migration, Literacy, and Healthcare Accessibility in Immunization Coverage of Banda District in the Bundelkhand Region of Uttar Pradesh, India

**DOI:** 10.7759/cureus.109494

**Published:** 2026-05-23

**Authors:** Lal Divakar Singh, Mohd Maroof, Suneel Kumar Kaushal, Shailendra Singh Chaudhary, Jalaj Chaturvedi

**Affiliations:** 1 Community Medicine, Rani Durgavati Medical College, Banda, Banda, IND

**Keywords:** diphtheria, immunisation gaps, migration, outbreak, qualitative research, vaccine hesitancy

## Abstract

Background: During August-September 2024, a diphtheria outbreak involving 10 children was reported from Devkher village of Naraini Block, Banda district, Uttar Pradesh, India. Despite the availability of diphtheria-containing vaccines under India's Universal Immunization Program, immunization gaps continue to persist in underserved rural communities.

Objective: To explore the social, geographic, and systemic factors contributing to immunization gaps during the diphtheria outbreak in Devkher village.

Materials and methods: A mixed-methods qualitative exploratory study was conducted between September and October 2024 in Devkher village. In-depth interviews were conducted among caregivers of affected children and frontline healthcare workers, including auxiliary nurse midwives (ANMs) and accredited social health activists (ASHAs). Descriptive outbreak-related data were obtained from local health records. Field observations and community-level contextual assessments were performed to understand geographic accessibility and healthcare outreach barriers. Thematic analysis was used for qualitative data interpretation.

Results: Major themes identified included migration-related disruption of vaccination schedules, poor awareness regarding immunization and booster doses, misinformation, inadequate documentation of vaccination status, and mistrust toward the healthcare system. Peripheral habitation, distance from healthcare facilities, and limited outreach services were identified as important contextual barriers contributing to delayed immunization and healthcare utilization.

Conclusion: Immunization gaps observed during the outbreak were influenced by interconnected social, geographic, and systemic factors. Strengthening outreach among migratory populations, improving immunization tracking systems, and enhancing community trust and healthcare accessibility are essential to prevent future outbreaks.

## Introduction

Diphtheria is an acute bacterial infection caused by *Corynebacterium diphtheriae* and remains a significant public health concern despite being a vaccine-preventable disease. It predominantly affects children under 15 years of age and is characterized clinically by sore throat, fever, and the formation of a pseudo-membrane over the tonsils and pharynx. If not treated promptly, it can lead to severe complications, such as airway obstruction, myocarditis, neuropathy, and death. Although effective vaccines and established global control strategies exist, diphtheria continues to persist in pockets of under-immunized populations, particularly in low- and middle-income countries [1].

Globally, diphtheria incidence has declined substantially following widespread immunization; however, sporadic outbreaks continue to be reported from regions with inadequate vaccination coverage. According to the World Health Organization (WHO), more than 8,600 cases were reported worldwide in 2021, with India contributing a significant proportion, making it one of the highest-burden countries for diphtheria [2]. Similar trends have been observed in other developing regions, where gaps in routine immunization and booster doses have resulted in periodic resurgence of the disease [3]. In India, the diphtheria-containing vaccine is administered under the Universal Immunization Program (UIP), which provides a three-dose primary schedule of pentavalent vaccine (DPT-HepB-Hib), followed by booster doses of diphtheria, pertussis, and tetanus (DPT) at 16-24 months and five to six years, and Td vaccine at 10 and 16 years [4]. Despite this structured program, gaps remain in booster dose coverage and continuity of immunization services. Studies have shown that waning immunity due to missed booster doses is a major contributor to diphtheria outbreaks among older children and adolescents [5].

Data from the National Family Health Survey (NFHS-5, 2019-21) indicate that, while 76.4% of children aged 12-23 months in India are fully immunized, significant regional disparities persist, particularly in northern states such as Uttar Pradesh [6]. In underserved regions such as the Bundelkhand area of Uttar Pradesh, factors such as socioeconomic deprivation, low maternal education, poor healthcare accessibility, and seasonal migration contribute to incomplete immunization and dropout from scheduled doses [7,8]. These contextual factors create vulnerable pockets where vaccine-preventable diseases such as diphtheria can re-emerge. In September 2024, a cluster of diphtheria cases was reported from Devkher village in the Naraini Block of Banda district, Uttar Pradesh, India. Among the 10 identified cases, seven were laboratory-confirmed, and three resulted in fatalities. Notably, eight out of the 10 cases lacked documented immunization history, highlighting critical gaps in vaccine coverage and health system outreach.

While surveillance data and outbreak reports provide valuable epidemiological insights, they often fail to capture the underlying socio-cultural and systemic factors influencing immunization behavior. Qualitative research plays a crucial role in understanding these community-level barriers, including perceptions of vaccines, health-seeking behavior, and trust in healthcare services [9]. Therefore, the present study adopts a mixed-methods approach, integrating qualitative exploration with descriptive outbreak data, to better understand the determinants of immunization gaps and to inform targeted public health interventions in similar high-risk settings.

Objective

The objectives of this study were as follows: (1) quantitatively assess the reported diphtheria cases during the outbreak in Devkher village, Banda district, Uttar Pradesh; (2) qualitatively explore the social, structural, and systemic factors contributing to missed immunization in the affected community; and (3) identify key policy and programmatic areas for strengthening immunization services and preventing future outbreaks.

## Materials and methods

Study design

This study employed a qualitative exploratory case study design based on the premise that knowledge is co-constructed through interactions between researchers and participants, reflecting their lived experiences. To strengthen the validity of findings, a mixed-methods approach was subsequently incorporated, allowing triangulation of qualitative insights with descriptive outbreak data and community mapping.

Study setting

The study was conducted in Devkher village, situated in the Naraini Block of Banda district, Uttar Pradesh, India. This rural and underserved area is characterized by poor health indicators and high rates of seasonal migration. The village has a population of 2,960 and is served by a primary health center (PHC) located in Badausa, approximately 7 km away, along with a subcenter in Pauhar at a distance of about 3 km. Devkher village was identified as the epicenter of a diphtheria outbreak during August-September 2024, reporting a total of 10 cases, including seven laboratory-confirmed cases and three deaths.

Participant selection and sampling

For the quantitative component, all 10 reported diphtheria cases, both suspected and confirmed, were included in the study. For the qualitative component, purposive sampling was employed to select participants directly relevant to the outbreak and immunization services. Primary participants consisted of caregivers (n=7) of children who were either infected or deceased due to diphtheria. Secondary participants included frontline health workers responsible for service delivery in the area, comprising one auxiliary nurse midwife (ANM) and two accredited social health activists (ASHAs). Families were included if they had a child affected by diphtheria during the outbreak, were residents of Devkher village, and were available and willing to participate. Health workers were included if they were actively involved in immunization or public health outreach in the affected area. Individuals unwilling to participate were excluded from the study.

Data collection

Data collection was carried out between September and October 2024, soon after the peak of the outbreak. Multiple data sources were utilized to ensure methodological triangulation. In-depth semi-structured interviews were conducted with caregivers to explore their experiences regarding the illness, immunization history, healthcare access, and perceptions of the health system. Health workers were interviewed using a structured guide focusing on operational challenges, vaccine delivery practices, record-keeping, and communication with the community. All interviews were conducted in Hindi, audio-recorded with verbal consent, and supplemented with detailed field notes. Each interview lasted approximately 15-30 minutes and was subsequently translated into English for analysis.

In addition, field observation and community mapping were undertaken in collaboration with health workers. A map of Devkher village was developed to identify key landmarks, such as Anganwadi centers, schools, temples, and health facilities, and to locate the households of affected children. Observations related to housing conditions, sanitation, and proximity to healthcare services were documented to assess environmental and spatial determinants. Furthermore, case registry data were obtained from local health records in the form of a structured line list of all 10 reported cases. This dataset included variables such as age, sex, clinical symptoms, immunization status, outcomes, and contact history.

Data analysis

Qualitative data obtained from interviews were analyzed using thematic analysis as described by Braun and Clarke. Transcripts were coded, and recurring themes were identified to understand barriers to immunization and healthcare access. Quantitative data from the case registry were analyzed using descriptive statistics, including age distribution, immunization status, and outcomes. Visual representations, such as tables, bar charts, and pie diagrams, were generated using Microsoft Excel (Microsoft® Corp., Redmond, WA) and Python. The community map was further utilized to interpret the spatial clustering of cases and the accessibility of health services.

Ethical considerations

Ethical approval for the study was obtained from the Institutional Ethics Committee of Rani Durgavati Medical College, Banda (IEC/RDMC/Cert/42). Informed verbal consent was obtained from all participants prior to data collection. Special care was taken to maintain the confidentiality and anonymity of participants. Emotional sensitivity was ensured while interviewing bereaved families, and interviews were paused or discontinued if participants showed signs of distress.

## Results

Geographic context

The affected households were predominantly clustered in peripheral hamlets of Devkher village, located more than 1 km from the nearest subcenter and Anganwadi outreach point, while the PHC serving the village was situated approximately 7 km away in Badausa. Poor road connectivity and limited transportation facilities contributed to reduced accessibility to routine immunization and healthcare services.

Overview of cases

Ten diphtheria cases were documented in Devkher village, Banda district. Of these, seven children were laboratory positive, of which three children died during the course of the outbreak. All cases occurred between August and October 2024, and most children were between the ages of 06 and 13 years, with the youngest being two years old. The first diagnosed case was a nine-year-old female reported on 15/08/2024, while the last case was a four-year-old female, which was reported on 1/10/2024. Both patients died within five days of symptom onset.

Table 1 shows that the majority of cases occurred among children aged 6-13 years, with one adolescent case aged 17 years and one toddler aged two years. A marked female predominance was observed, with 90% (9 out of 10) of the cases occurring in females. Immunization documentation was notably poor, as 90% (9 out of 10) of the affected children did not possess a vaccination card. The case fatality rate was 30% (3 out of 10), with all deaths occurring in children younger than 10 years. A history of contact with a confirmed case was present in 60% (6 out of 10) of the children, while 70% (7 out of 10) were laboratory-confirmed cases of diphtheria. Regarding immunization status, 50% (5 out of 10) of the children had received no doses of pentavalent, DPT, or tetanus-diphtheria vaccines, 30% (3 out of 10) had received incomplete vaccination for their age, and only 20% (2 out of 10) were fully immunized as per the age-appropriate schedule.

**Table 1:** Summary of the reported diphtheria cases

Case No.	Age (yrs)	Sex	Vaccination Card	Outcome of cases	Contact History	Lab Result	Dosage of vaccines received (Penta/DPT/Td)
1	9	Female	No	Death	Yes	Positive	0
2	4	Female	No	Discharged	Yes	Positive	1
3	11	Female	No	Discharged	Yes	Positive	3
4	17	Female	No	Discharged	No	Positive	0
5	10	Female	No	Discharged	Yes	Negative	2
6	11	Female	No	Discharged	No	Negative	0
7	8	Male	No	Discharged	Yes	Negative	0
8	2	Female	No	Death	No	Positive	0
9	4	Female	Yes	Discharged	Yes	Positive	4
10	4	Female	No	Death	No	Positive	4

**Figure 1:**
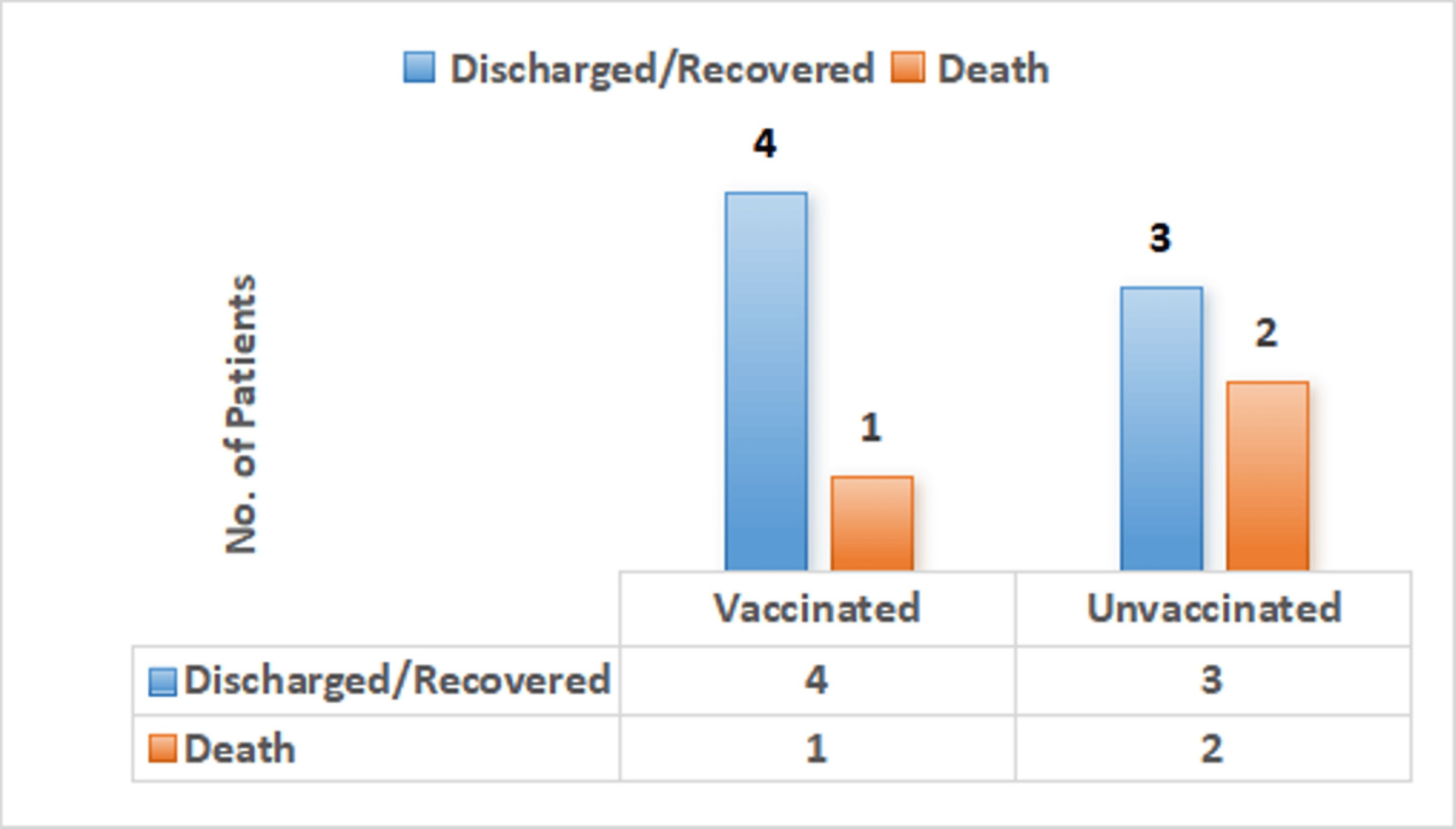
Relation of vaccination status and recovery outcome

Figure 1 shows that 50% of the patients were vaccinated and 50% were unvaccinated. Among the five vaccinated cases, only two (40%) were fully vaccinated, while three (60%) were incompletely vaccinated for their age. Among the vaccinated children, four (80%) recovered, and one died. In contrast, among the unvaccinated children, three (60%) recovered, while two died. This suggests that children with vaccination, either full or partial, had better outcomes than unvaccinated children with diphtheria during the present outbreak. However, the death of one fully vaccinated four-year-old child raises concerns about other possible issues, such as vaccine effectiveness, storage/transport conditions, and individual health factors.

Thematic analysis: community interviews

The thematic analysis of qualitative interviews revealed five major themes influencing immunization gaps during the diphtheria outbreak. These themes are interrelated and highlight the complex interplay between socioeconomic status, health system trust, migration, and frontline service delivery.

Theme 1: Migration as a Disruptor of Routine Immunization

Migration is the most dominant structural barrier to trade. Families frequently migrated seasonally for agricultural or labor work (e.g., to Punjab, Delhi, and Bhopal), disrupting continuity of care and access to routine health services. Due to their absence during scheduled immunization days, children missed critical vaccine doses, especially DPT/Td boosters.

We go for work for eight months. There, no one comes for vaccination and when we return, no one follows us up here. - Father of case number 10 (Table 1)

In several cases, children died despite returning to their village every year, reflecting a failure of the system to track and follow up on migratory children.

Theme 2: Low Health Literacy and Awareness of Immunization Schedule

Caregivers demonstrated limited knowledge about the immunization schedule, the diseases vaccines prevent, or the importance of booster doses. Many believed that a few early life vaccines were sufficient and were unaware of the role of DPT in preventing diphtheria specifically.

We thought vaccines are for babies only... no, one told us about giving it again at school age. - Mother of case number 2

This indicates a gap in community-based health education, particularly in contexts where children are not enrolled in school. None of the three deceased children in this study was a school-goer, reinforcing the role schools play in health promotion.

Theme 3: Documentation Manipulation and Post-Facto Record Filling

A critical theme was the retrospective filling or fabrication of vaccination records after the outbreak began. Several families reported that immunization cards were issued or filled out only after their child had died or fallen seriously ill. This practice further eroded trust in public health services and prevented timely intervention.

They gave the vaccine card after my child died... they just filled it up to save themselves. - Mother of case number 1

This practice reflects accountability failures within the local health workforce. ANM interviews revealed that missing cards were sometimes "reconstructed" based on memory or secondary reports - contradicting WHO's emphasis on traceable, real-time data in outbreak control (WHO, 2022) [2]. Despite several serious attempts, real-time data from U-win or other web portals were not made available to us by the health authorities for the study.

Theme 4: Mistrust and Interpersonal Conflict With Health Workers

Tensions between families and community health workers (ASHAs and ANMs) were recurrent. Several caregivers expressed mistrust, frustration, or anger, alleging negligence or poor communication.

The ASHA came once and said to come to the Anganwadi, but there was no vaccine. Now they say it’s our fault. - Father of case number 8

This breakdown in trust led to emotional confrontations, with investigators at times having to separate ASHAs from bereaved families during interviews. The absence of respectful dialogue and transparent communication contributed to vaccine hesitancy and non-compliance.

Theme 5: Structural and Operational Weakness in Service Delivery

Frontline workers themselves acknowledged chronic logistical problems-vaccine stockouts, irregular supply, and absence of cold-chain monitoring in remote locations. ANMs often received vaccines on the same day as the administration, limiting their capacity to conduct pre-planned sessions.

Sometimes, the vaccine does not come till the afternoon. We can’t keep going back to every house. - ANM Interview

Health staff lacked a follow-up system and had no interest in tracking defaulters, especially among migrant families. Outreach was limited to static immunization days, with no follow-up mechanisms for absentee households.

This detailed thematic analysis reinforces the need for trust-based community engagement, accountable documentation, and mobile outreach strategies that adapt to the lived realities of migrant and vulnerable populations. It also demonstrates how frontline health staff are constrained by broader systemic limitations.

Interview with ANM

Table 2 reveals that ANM had adequate knowledge of cold chain maintenance and was aware of the open vial policy. Vaccines were supplied to her by the auxiliary vaccine delivery (AVD) worker and transported to the field site using ice packs to maintain appropriate temperature conditions. The ANM reported that fever is a commonly observed adverse event following DPT vaccination. However, challenges were noted in documentation practices, as immunization cards were sometimes lost or filled out retrospectively. Regarding vaccine management, any leftover doses were returned to the cold chain handler to ensure proper storage and minimize wastage.

**Table 2:** Auxiliary nurse midwife (ANM) interview summary AVD: auxiliary vaccine delivery; DPT: diphtheria, pertussis, and tetanus

Parameter	Response
Cold Chain Knowledge	Yes
Open Vial Policy Awareness	Yes
Vaccine Transport Method	Supplied by an AVD worker
Vaccine Storage at the Field Site	Carried in ice packs
Adverse Events After DPT	Fever frequently reported
Documentation Issues	"Sometimes cards are lost or filled later."
Leftover Dose Management	Sent back to the cold chain handler

## Discussion

The findings of this study underscore several key factors contributing to the diphtheria outbreak in Devkher village of Naraini Block in Banda district, Uttar Pradesh. The occurrence of 10 cases with three deaths within a single village indicates a localized yet potentially preventable outbreak driven by structural and systemic gaps in the health system. The clustering of cases in geographically peripheral areas highlights the importance of spatial determinants of disease transmission. A similar pattern was reported by Bitragunta et al., who demonstrated that diphtheria cases in India were concentrated in underserved regions with limited healthcare access, comparable to the clustering observed in the present study [10]. Similar geographic clustering of vaccine-preventable diseases has also been documented in rural settings with limited service outreach and accessibility barriers [11].

A major finding of this study was that 80% of the cases were not fully immunized, indicating a substantial immunization gap. This is consistent with the findings of Murhekar, who reported that incomplete or absent vaccination remains the most significant risk factor for diphtheria outbreaks in India [5]. Similarly, the World Health Organization reported that the resurgence of diphtheria globally is strongly associated with gaps in routine immunization and missed booster doses [12]. Studies from South-East Asia have also emphasized that failure to complete booster doses leads to waning immunity and increased susceptibility among older children [13]. In the present study, although severe outcomes were observed in both vaccinated and unvaccinated children, most deaths occurred among unvaccinated individuals, which aligns with global evidence showing that vaccination significantly reduces mortality risk [14]. However, interpretation regarding broader immunization coverage trends at the village level was limited due to the unavailability of complete historical immunization coverage records for the preceding years.

The demographic pattern in this study showed a predominance of female cases (90%). However, this observation should be interpreted cautiously because gender-wise attack rates could not be calculated due to the non-availability of denominator population data for the pediatric population in the village. Although similar sex disparities have been reported in rural settings, the present study was not designed to establish gender-based differences in susceptibility or immunization practices. Singh et al. observed that social and economic inequalities, including gender-related disparities, can influence immunization uptake [15]. Additional studies from rural India have also reported that gender bias and preferential healthcare-seeking for male children can indirectly affect immunization coverage among females [16].

Migration emerged as one of the most significant disruptors of routine immunization in the present study. Families migrating for seasonal labor often miss scheduled vaccinations, particularly booster doses. This finding is strongly supported by Singh et al., who identified migration and socioeconomic factors as important determinants of under-immunization [15]. Furthermore, global evidence from the WHO demonstrates that population mobility disrupts the continuity of immunization services and contributes to outbreaks of vaccine-preventable diseases [12]. Studies from similar low-resource settings have also highlighted that migratory populations often fall outside routine health system tracking mechanisms, leading to missed opportunities for immunization [17]. The absence of a structured tracking system for migratory populations in the present study reflects a systemic gap consistent with these findings.

Another important observation was poor documentation of immunization status, with many children lacking vaccination cards or having records completed retrospectively. This reflects weaknesses in health system accountability and data management. Comparable findings were reported by Mohanty et al., who highlighted that inadequate documentation and unreliable immunization records significantly impair outbreak response and surveillance [18]. The WHO emphasizes that accurate and real-time data recording is essential for effective immunization programs and outbreak control [2]. Additional studies have also noted that weak health information systems contribute to underreporting and delayed identification of outbreaks [19]. The study also revealed mistrust between caregivers and frontline health workers, which contributed to reduced healthcare utilization. Similar findings have been reported in Indian and global studies, where poor communication, misinformation, and lack of trust were identified as key barriers to immunization uptake [9]. Strengthening interpersonal communication and community engagement has been shown to significantly improve vaccine acceptance and coverage [20].

Overall, the findings of this study suggest that diphtheria outbreaks are not solely due to a lack of vaccine availability but are driven by a complex interplay of social, behavioral, and systemic factors. Addressing these issues requires strengthening health system accountability, improving documentation practices, enhancing outreach to migrant populations, and rebuilding community trust. In addition, strengthening surveillance systems and ensuring timely booster coverage are essential to prevent future outbreaks. Without addressing these root causes, immunization programs are likely to continue falling short in vulnerable populations, thereby sustaining the risk of vaccine-preventable disease outbreaks.

Limitations

The present study had certain limitations. The study was conducted in a single village with a small number of reported diphtheria cases, which limits the generalizability of the findings. Gender-wise attack rates and village-level immunization coverage trends could not be calculated due to the unavailability of denominator population data and complete historical immunization records. The qualitative findings were based on participant recall and may therefore be subject to recall bias. In addition, the exploratory nature of the study and the absence of a comparison group limited the ability to establish causal associations between identified factors and the outbreak.

Key messages

Migration, poor awareness, misinformation, and inaccessibility of healthcare contributed to immunization gaps; trust-building and targeted outreach are urgently needed.

## Conclusions

The diphtheria outbreak in Devkher village highlights critical gaps in routine immunization coverage and health system functioning in an underserved rural setting. A high proportion of children were either unvaccinated or partially immunized, with migration, poor awareness, and weak follow-up mechanisms contributing significantly to missed vaccinations. Geographic inaccessibility and deficiencies in documentation further hindered timely outbreak response and verification of immunization status. Following identification of the outbreak, intensified immunization activities, active surveillance, and outreach sessions were conducted in the affected area; however, the outbreak underscored the need for sustained preventive strategies beyond reactive measures. The findings emphasize that improving vaccine availability alone is insufficient without strengthening outreach, surveillance, and accountability mechanisms. Targeted strategies focusing on migratory populations, community engagement, and reliable immunization tracking systems are essential to address these issues. Addressing these systemic and social determinants is crucial for preventing similar outbreaks in vulnerable populations in the future.

## Appendices

Appendix 1: Semi-structured interview questionnaire

Section A: Identification Details

(To be filled by interviewer)

Participant ID: ______

Type of participant: Caregiver / Health worker

Name (optional): ______

Age: ______ years

Sex: Male / Female

Village: ______

Date of interview: ______

Section B: Caregiver Interview (Parents/Guardians of Affected Child)

1. Child Health and Illness History

Can you describe the illness your child experienced?

When did the symptoms start?

What symptoms did your child have (fever, sore throat, breathing difficulty, etc.)?

Did you seek treatment? If yes, where and when?

2. Immunization Status

Do you have your child’s immunization card? (Yes/No)

If yes, can you show it? If no, why is it not available?

Do you know which vaccines your child has received?

Was your child vaccinated regularly as per schedule?

If not, what were the reasons for missing vaccination?

3. Awareness and Knowledge

What do you know about vaccines and the diseases they prevent?

Do you know that vaccines are required after infancy (booster doses)?

Has any health worker explained the vaccination schedule to you?

4. Migration and Mobility

Does your family migrate for work? (Yes/No)

If yes, where do you go and for how long?

During migration, does your child receive vaccines?

Did migration affect your child’s vaccination schedule?

5. Access to Health Services

How far is the nearest health facility from your home?

Do health workers visit your home regularly?

Have you ever faced difficulty in reaching vaccination services?

Were vaccines always available when you visited the center?

6. Interaction with Health Workers

How often do ASHA/ANM workers visit your home?

Do they inform you about vaccination days?

Do you trust the health workers? Why or why not?

Have you ever had any disagreement or issue with them?

7. Documentation and Record-Keeping

When was your child’s vaccination card issued?

Was it filled regularly or later?

Has any information been added after your child became ill?

8. Perceptions and Beliefs

Do you think vaccines are necessary for children?

Are there any fears or myths in your community about vaccines?

What do people in your village generally think about vaccination?

9. Suggestions

What do you think can be done to improve vaccination in your village?

What support do families like yours need?

Section C: Health Worker Interview (ANM/ASHA)

What is your role in immunization services?

How many households/children are you responsible for?

How are vaccination sessions conducted in your area?

Are vaccines regularly available?

Do you face any logistical challenges?

Are you trained in cold chain maintenance?

How are vaccines transported to your area?

How do you ensure proper storage at field sites?

What do you do with leftover vaccine doses?

How do you inform families about vaccination sessions?

Do families cooperate with immunization activities?

What challenges do you face in convincing caregivers?

Do families in your area migrate frequently?

How do you track children who migrate?

What happens if a child misses vaccination?

How do you maintain immunization records?

Do you face issues with missing or incomplete records?

Are records ever updated retrospectively?

What common side effects are reported after vaccination?

How do you manage adverse events?

What are the biggest challenges in your work?

Do you receive adequate support from the health system?

What improvements are needed in immunization services?

## References

[1] (2026). Diphtheria. https://www.who.int/news-room/fact-sheets/detail/diphtheria.

[2] (2026). Diphtheria reported cases and incidence. https://immunizationdata.who.int/pages/incidence/diphtheria.html.

[3] Clarke KE, MacNeil A, Hadler S, Scott C, Tiwari TS, Cherian T (2019). Global epidemiology of diphtheria, 2000-2017. Emerg Infect Dis.

[4] (2026). Universal Immunization Programme (UIP) Operational Guidelines. New Delhi: MoHFW. New Delhi: MoHFW.

[5] Murhekar MV (2020). Resurgence of diphtheria in India. J Infect.

[6] (2026). National Family Health Survey (NFHS-5) - 2019-21. Mumbai: IIPS.

[7] Singh PK, Rai RK, Alagarajan M, Singh L (2012). Determinants of maternity care services utilization among married adolescents in rural India. PLoS One.

[8] Gupta P, Prakash D, Srivastava JP (2015). Determinants of immunization coverage in Lucknow district. N Am J Med Sci.

[9] Larson HJ, Jarrett C, Eckersberger E, Smith DM, Paterson P (2014). Understanding vaccine hesitancy around vaccines and vaccination from a global perspective: a systematic review of published literature, 2007-2012. Vaccine.

[10] Bitragunta S, Murhekar MV, Hutin YJ, Penumur PP, Gupte MD (2008). Persistence of diphtheria, Hyderabad, India, 2003-2006. Emerg Infect Dis.

[11] Nath B, Singh JV, Awasthi S, Bhushan V, Kumar V, Singh SK (2007). A study on determinants of immunization coverage among 12-23 months old children in urban slums of Lucknow district, India. Indian J Med Sci.

[12] (2026). World Health Organization. Diphtheria vaccine: WHO position paper. Wkly Epidemiol Rec. 2017;92(31). https://www.who.int/publications/i/item/diphtheria-vaccines-who-position-paper-august-2017.

[13] (2026). World Health Organization vaccination coverage cluster surveys: reference manual. https://iris.who.int/items/73509e3f-0fcd-4295-b92c-bcf25c5c073c.

[14] Vitek CR, Wharton M (1998). Diphtheria in the former Soviet Union: reemergence of a pandemic disease. Emerg Infect Dis.

[15] Singh A, Pallikadavath S, Ram F, Alagarajan M (2014). Do antenatal care interventions improve neonatal survival in India?. Health Policy Plan.

[16] Pande RP, Yazbeck AS (2003). What's in a country average? Wealth, gender, and regional inequalities in immunization in India. Soc Sci Med.

[17] Gera T, Shah D, Garner P, Richardson M, Sachdev HS (2016). Integrated management of childhood illness (IMCI) strategy for children under five. Cochrane Database Syst Rev.

[18] Mohanty P, Satpathy SK, Patnaik S, Patnaik L (2021). Out-of-pocket expenditure and its predictors for illness of under-five children: a cross-sectional study in urban slums of eastern India. J Family Med Prim Care.

[19] Bosch-Capblanch X, Ronveaux O, Doyle V, Remedios V, Bchir A (2009). Accuracy and quality of immunization information systems in forty-one low income countries. Trop Med Int Health.

[20] Dubé E, Laberge C, Guay M, Bramadat P, Roy R, Bettinger J (2013). Vaccine hesitancy: an overview. Hum Vaccin Immunother.

